# Supplementary material for: A Transcriptional Activator of Ascorbic Acid Transport in Streptococcus pneumoniae Is Required for Optimal Growth in Endophthalmitis in a Strain-Dependent Manner
Source: Microorganisms. 2019 Aug 24;7(9):290. doi: 10.3390/microorganisms7090290 (PMC6780617; doi:10.3390/microorganisms7090290)
Supplement: Supplementary file 1 [file microorganisms-07-00290-s001.zip › microorganisms-572813-proofreading sup/Supplemantary Figures.pdf]

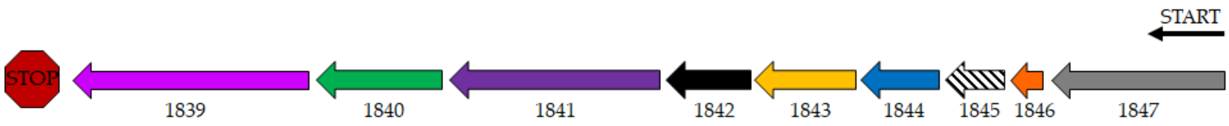

Figure S1. The *ula* operon in *S. pneumoniae* as determined by Afzal et al. [29]. Gene identifier numbers are for strain D39. *Spd_1846* was identified as having reduced fitness in vitreous humor.

```
Query  481      AGTGAGTTGCTGGAATCCTCCATAGGGATTTGGGATTTGGATTATGTTTTATCCAGTTGG  540
                |||||||||||||||||||||||||||||||||| |||||||||||||||||||||||||
Sbjct  2104171  AGTGAGTTGCTGGAATCCTCCATAGGGATTTGGGGTTTGGATTATGTTTTATCCAGTTGG  2104112

Query  601      CAACTAGTACCTATTGTTAATCAATTAAAAGTTTGCCTTTTCGGTTTAGTATTCATTCTG  660
                || ||||||||||||||||||||||||||||||| |||||||||||||||||||||||||
Sbjct  2104051  CAGCTAGTACCTATTGTTAATCAATTAAAAGTTTACCTTTTCGGTTTAGTATTCATTCTG  2103992

Query  781      GTGTTTTCTGAGGATGATTATCGCTATATCACAGTTTATTATCAAGTTGCTTTGAAGGT  840
                |||||||||||||||||||||||||||||||| |||||||||||||||||||||||||||
Sbjct  2103871  GTGTTTTCTGAGGATGATTATCGCTATATCACCGTTTATTATCAAGTTGCTTTGAAGGT  2103812

Query  1261     CCTCAAATTGAGTTTATAGGAACCTCAAAGATTGATGATTTACAGGTGAAAGCTAGTAGT  1320
                |||||||||||||||||||||||||||||||||||||||||||||||||||| |||||||
Sbjct  2103391  CCTCAAATTGAGTTTATAGGAACCTCAAAGATTGATGATTTACAGGTGAAAGTTAGTAGT  2103332
```

Figure S2. Nucleotide differences between D39 and E335 in ulaR2. D39 is the Query line and E335 is the Subject.

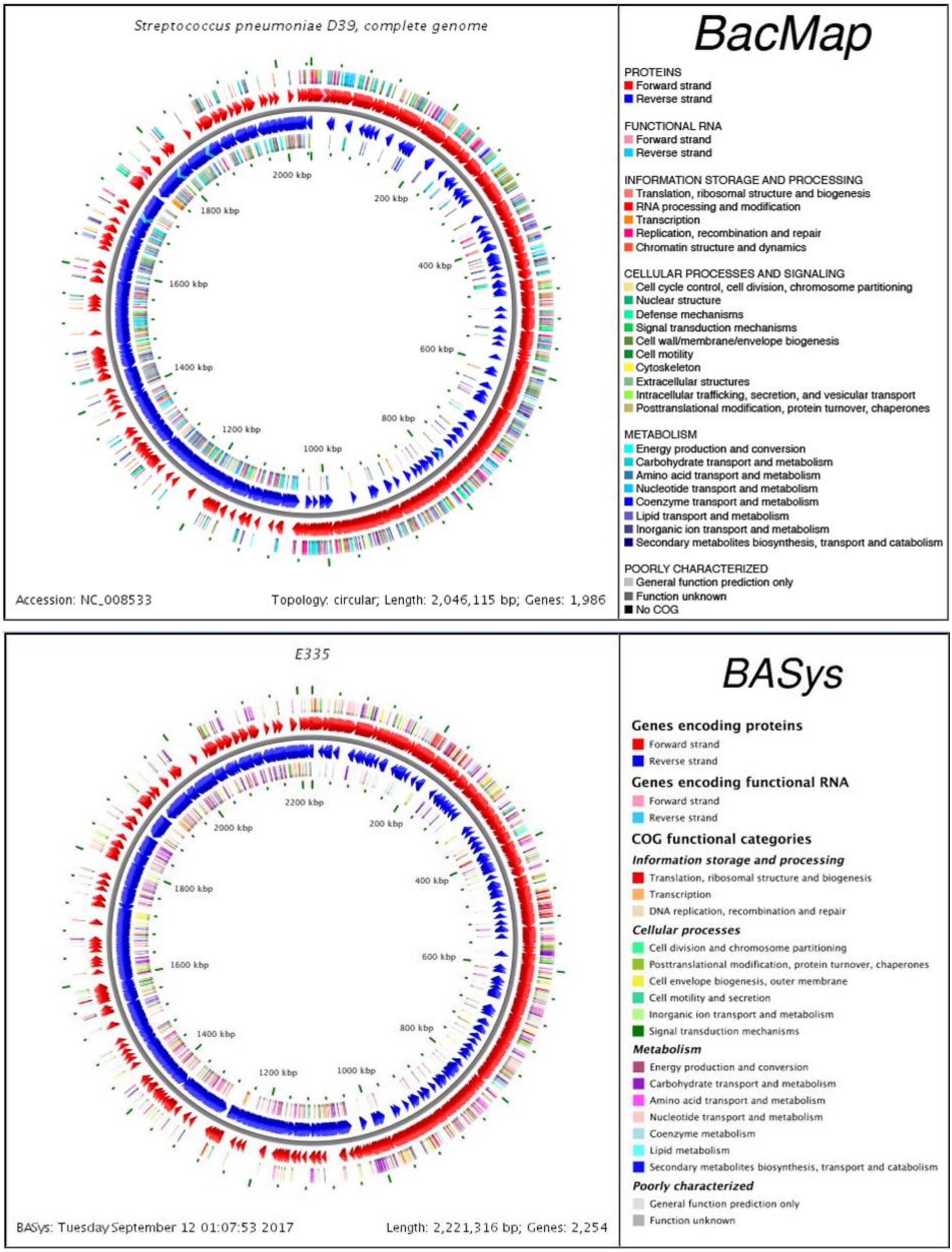

Figure S3. Genome maps of *S. pneumoniae* D39 generated by BacMap using GenBank accession #NC008533 (top panel) and E335 by BaSys using GenBank accession #CP026670 (bottom panel).
